# Informal Network Members’ Perspectives and Experiences on Work for People with Intellectual Disabilities: A Thematic Synthesis

**DOI:** 10.1007/s10926-023-10128-0

**Published:** 2023-07-08

**Authors:** Moniek A. C. Voermans, Ton Wilthagen, Petri J. C. M. Embregts

**Affiliations:** 1 Tranzo, Tilburg School of Social and Behavioral Sciences, Tilburg University, P/O Box 90153, Tilburg, 5000 LE The Netherlands; 2 Amarant, Healthcare Organisation for People with Intellectual Disabilities, Tilburg, The Netherlands; 3 Public Law and Governance, Tilburg Law School, Tilburg University, Tilburg, The Netherlands

**Keywords:** Daytime Activities, Employment, Informal Networks, Intellectual Disabilities, Work

## Abstract

**Purpose:**

The level of participation of people with intellectual disabilities (ID) in various forms of work, including daytime activities, appears to be suboptimal. Informal networks of people with ID constitute crucial forms of support, as they can significantly influence occupational choices and opportunities. This review aims to synthesize existing research for the purpose of examining how informal network members perceive the meaning of employment or daytime activities for their relatives with ID.

**Methods:**

Following the PRISMA guideline, a systematic search of scientific literature published between 1990 and July 2022 was conducted. The qualitative results from twenty-seven studies (qualitative and mixed-method) were analyzed using thematic synthesis.

**Results:**

Four overarching themes and several subthemes were identified: (I) Ensuring customized work for my relative; (II) The ongoing need to collaborate and share care responsibilities with professionals; (III) The meaning of work for both my relative and myself; and (IV) Achieving full work participation for my relative is neither straightforward nor self-evident.

**Conclusions:**

Informal networks place great value upon customized and sustainable work opportunities for their relatives with ID, particularly community-based work. While network members play an important role in creating these opportunities, they encounter obstacles resulting from both collaboration difficulties with professionals and employers and public and structural forms of stigma. Researchers, professionals, policy makers, and employers are encouraged to collaborate with individuals with ID as well as their networks to increase the meaningful work opportunities available to them.

**Supplementary Information:**

The online version contains supplementary material available at 10.1007/s10926-023-10128-0.

## Background

Social changes in Western societies over the course of the twentieth century have led to a shift away from collectivist values towards more individualistic values characterized by individuals’ rights and self-determined choices [1]. In line with these social changes, many Western countries also pursued policies focused on the integrated living of people with ID, and the recognition of their equal rights to citizenship, as a substitute for living and working in large-scale institutions, often outside or at the border of cities [2, 3]. These major transformations, which include, among other things, deinstitutionalization, have hitherto regrettably not resulted in equal opportunities in society for people with ID [1, 4].

Participation in employment represents an important pathway through which to stimulate equal rights and full participation in society for people with ID [5–7]. However, despite evidence and policies in many countries aimed toward promoting inclusion within the workforce for people with disabilities [8, 9], the employment rates for people with disabilities remain significantly lower compared to the general population [8, 10, 11]. Alongside the small proportion of people with ID who participate in competitive employment, people with ID also participate in a wide range of employment or daytime activities that can take place in either sheltered or integrated work environments [12]. Sheltered employment is defined by Metzel et al. (2007, p. 51) as “employment in a facility where most people have disabilities with ongoing work-related support and supervision” and can take place in sheltered workshops, day centers and vocational rehabilitation centers [12, 13]. Integrated employment is defined by Migliore et al. (2007, p. 7) as “taking place in a community setting in the general labor market where the proportion of workers with disabilities does not exceed the natural proportion in the community” and can take the form of competitive employment, supported employment, entrepreneurship, or self-employment [12, 14]. Unfortunately, the participation rates for people with ID in both integrated and sheltered employment remain suboptimal [15].

People with ID themselves appear to perceive participation in integrated work activities as incredibly valuable, as far as it enables them to make a meaningful contribution to society [7, 16, 17]. They also appreciate the opportunity to do work activities in a sheltered environment, because it affords them opportunities for both engaging in social relationships and growth and development [18–20]. Successfully identifying which type of work best suits a particular person with ID depends on numerous factors, such as person-level attributes, individual needs and goals, skills, educational preparation, social opportunities, societal values, and family and community support [6]. Parents and other members of the informal network of people with ID constitute a potentially important resource in terms of identifying and sustaining suitable work options [21, 22], insofar as people with ID might need more support than people without ID to make self-determined choices [23]. Therefore, the present systematic review focuses on informal network members’ perspectives on employment or daytime activities for their relatives with ID.

People with ID can be supported in making self-determined choices by professionals as well as by people from their informal network, such as parents, relatives or friends and peers. The forms of support provided by informal networks to people with ID display particular characteristics. For example, although the social networks of people with ID frequently appear to be small and largely composed of family members and professionals [24, 25], they are often characterized by a high frequency of contact [26]. In many cases, parents provide most of the practical [27] and emotional [24] forms of support for their offspring with ID, but people with ID also assign important roles to others in their family and broader informal network, such as siblings and friends [24, 28].

When people need to make self-determined choices, the social values of a person’s informal network may have a major influence on this process [29]. The values, beliefs and experiences shared by informal network members can also greatly influence people’s occupational choice [30]. Therefore, informal network members of people with ID – and their values, beliefs, and experiences – may contribute to how people with ID experience both the meaning of and opportunities for participation in employment [21, 22]. This may be especially so given the aforementioned reliance of people with ID on and their need for support from their informal network.

Consequently, informal networks may play an important role in the occupational choice of people with ID and, in turn, their participation in society via employment opportunities and job retention [21, 22]. Therefore, these informal networks are a vitally important resource for identifying appropriate opportunities for participation in suitable employment or daytime activities for people with ID, further enhancing their own self-determination. It is thus critically important to better understand informal network members’ perspectives (i.e., values, beliefs, and experiences) on the meaning of the broad spectrum of integrated and sheltered employment and daytime activities for people with ID. Given the scarcity of research on informal network members’ perspectives on this issue, this study sets out to synthesize existing research on these perspectives in a systematic review in order to answer the following research question:

How do informal networks perceive the meaning of participation in employment or daytime activities for their adult relatives with ID and their social environment?

## Method

In conducting and reporting on the systematic review, the guideline for Preferred Reporting Items for Systematic Reviews and Meta-Analysis [31] were implemented.

### Search Strategy

First, the Population, Intervention/exposure, Comparison and Outcome (PICO) approach [31] was used to determine the core elements for the systematic review. The Population (P) was defined as ‘informal network members of people with ID.’ The informal network was defined broadly, as far as it included family members as well as other (non-paid) network members who may be influential in the occupational choices of the person with ID, such as friends. This decision was based on the fact that people with ID themselves sometimes also consider non-relatives to be family members [24]. Intervention (I) was defined as ‘Participation in – paid or non-paid – employment or daytime activities by people with ID’, which, as aforementioned, includes all forms of sheltered and integrated employment. Due to the descriptive character of the research question, the Comparison (C) component was deemed to be not relevant for this study. Outcome (O) was defined as ‘Perspectives (i.e., values, beliefs, and experiences) of informal network members of people with ID toward employment and daytime activities.’ Initially, the O component was not specified in the search strategy, as the perspective of informal network members is an underexplored research area, and hence, any and all information about their perspectives was deemed to be of interest for this study. Subsequently, a search strategy was developed and conducted, based on the Exhausted Search Method [32], in collaboration with an experienced information specialist. Four databases (i.e., Embase, MEDLINE (Ovid), PsychINFO (Ovid), and Web of Science) were systematically searched for empirical, peer-reviewed articles, published in English between January 1, 1990, and July 5, 2022. Given that contemporary research on the employment of people with ID only started in 1990 [17], articles prior to the year 1990 were excluded. The full search strategy is provided in Appendix I.

### Study Selection

**Fig. 1:**
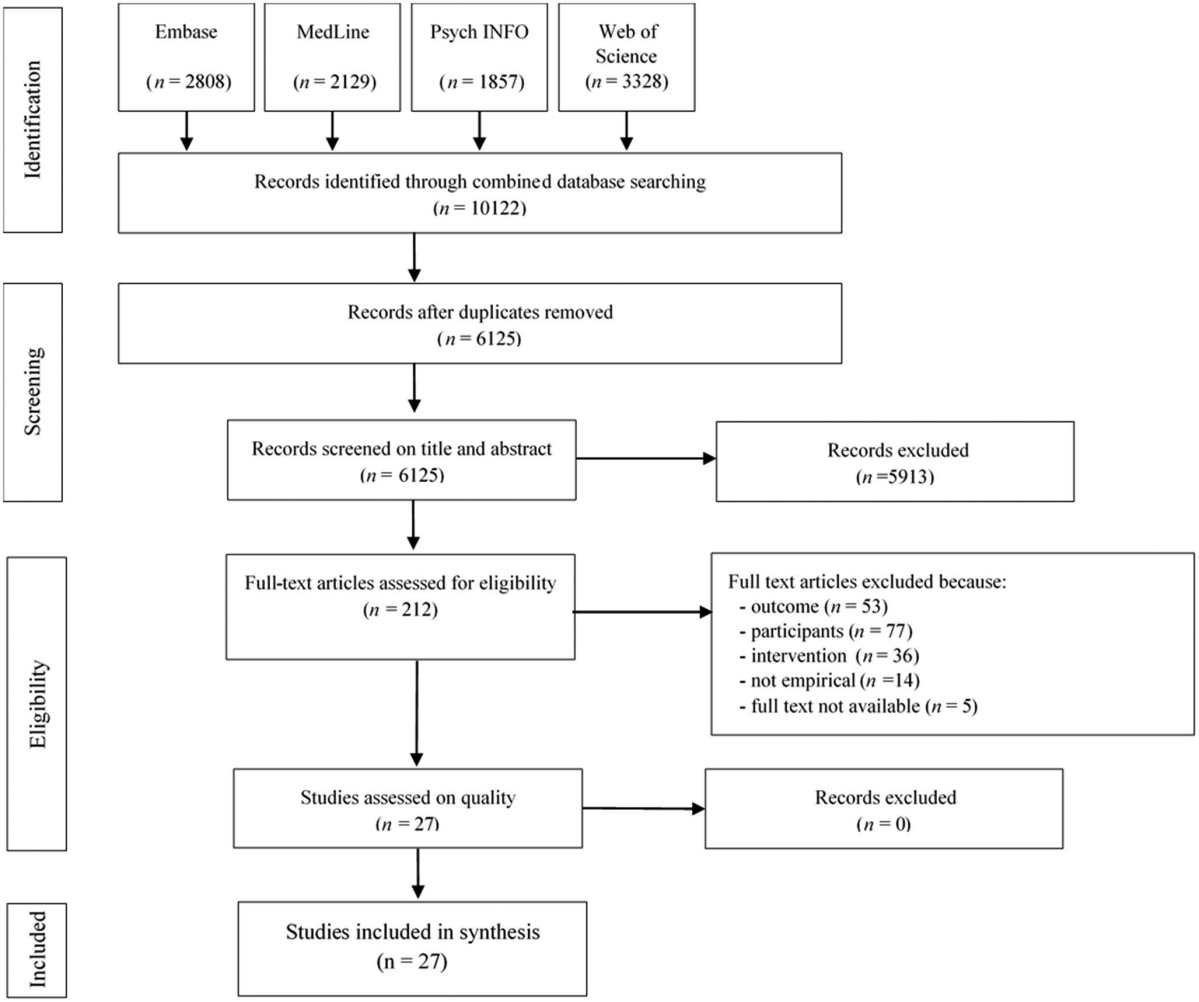
Flow-chart Study Selection

In accordance with the PRISMA guidelines [31], the selection process consisted of four phases: (I) identification, (II) screening, (III) eligibility, and (IV) inclusion. A flowchart of the selection process is shown in Figure 1. During the first phase, duplicates were removed, along with publications prior to 1990. The second phase comprised reviewing the 6125 titles and abstracts of all remaining articles for the purpose of inclusion or exclusion. In order to refine the inclusion and exclusion criteria, fifty publications were reviewed and discussed by the first author and a second researcher. The criteria for inclusion and exclusion are represented in Table 1. All publications were independently reviewed by both the first author and a second researcher to improve inter-researcher consistency. They agreed on 97% of all titles and abstracts. Any disagreements were discussed between both reviewers until a consensus was reached. In the event of uncertainty, the article in question was included and proceeded to the next phase. During the third phase (i.e., ‘eligibility’), the same researchers independently reviewed the full texts of the remaining articles (n = 212). During this phase, studies were also excluded according to the criteria concerning the Outcome (O), i.e., perspectives (values ,beliefs, and experiences) of informal network members concerning employment of people with ID. Based on this broad definition, only qualitative studies that properly captured the perspectives of the participants were included as well as mixed-methods studies whose qualitative component met this criterion. Quantitative studies were thus excluded at this point. The researchers agreed on 87% of the full texts. Any disagreements were discussed until a consensus was reached, and in the event of uncertainty a senior researcher was consulted. Subsequently, the quality of the eligible studies (n = 27) was assessed using the Mixed-Method Appraisal Tool (MMAT), version 2018 [33], which is applicable for the assessment of different types of research designs. The quality assessment was conducted in order to provide a proper assessment of the quality of the studies and any potential bias, and was conducted by the first author in close cooperation with a second researcher. The quality criteria for each study were discussed in detail until a full consensus was reached, which resulted in studies assessed as having high quality (n = 16), medium quality (n = 6) or low quality (n = 5). Information on the quality of the studies is included in Table 3. All of the authors frequently discussed the selection process and were consulted in the event of any uncertainties.

**Table 1:** Inclusion- and exclusion criteria

	Inclusion criteria	Exclusion criteria
General	• Empirical articles • Publications > 1990 • Articles in English	• Non-empirical articles, including systematic reviews • Publications < 1990 • Articles in other languages
Population (P)	Studies concerning: • (non paid) network members of adults with ID (e.g. parents, grandparens, siblings, friends, acquaintances, mentors) • (non paid) network members of adults with other comorbid disorders, frequently associated with ID (i.e. autism, down syndrome, epilepsy, cerebral palsy, FASD)	Studies concerning: • (paid) professional caregivers (e.g. support staff, psychologists, teachers, nurses) • People with ID themselves • Other populations with no reference to ID (e.g. psychiatric patients, ex-cancer patients, people with physical disabilities, autism clearly without ID, ADHD, SLI, DCD and ABI) • Infants and children (< 18 years) • Adults > 65 years • A mixed sample of participants where separate results could not be extracted for the subgroup meeting inclusion criteria
Intervention (I)	Studies concerning informal network members of: • Adults with ID participating in employment or daytime activities at present or in the past • This includes all forms of work or daytime activities, including activities as an expert by experience or co-researcher	Studies concerning: • Education / educational activities • The transition process from education to employment/daytime activities • The transition process from employment/daytime activities to retirement • Daily living • Leisure activities (e.g. sports or faith communities) • Therapy or therapeutic activities
Outcome (O)	• Experiences, values, beliefs and attitudes of informal network members	• Factual information from informal network members (e.g. the number of workplaces that their relative with ID has had) • Informal network members explicitly representing the perspective of their relative as a proxy (e.g. when they had answered behavioural questionnaires)

### Data Extraction and Analysis

Each study was read several times and summarized by the first author in order to familiarize herself with all the studies. For each study, specific information was extracted about the author, year of publication, country, aim(s), study design, participants (P), the employment situation of the relatives with ID (I), and the main results. In addition, given that not all of the results in the studies met the inclusion criteria, only data that met the inclusion criteria were extracted from the results sections of all the studies, in preparation for the thematic synthesis. Data extraction was conducted by the first author, while a second researcher independently extracted data from 20% of the studies (with a 94% level of agreement). Thematic Synthesis [34] was employed as a method for data analysis.

To start the thematic synthesis, the extracted data were carefully studied by the first author in order to familiarize herself selves with the data. Next, all data were coded line by line. Coding was conducted in a word processing program by the first author. The second researcher independently coded 20% of the data (with an 85% level of agreement). Both researchers then discussed any disagreements until a consensus was reached, and in the event of any uncertainty a senior researcher was consulted. Subsequently, themes and subthemes were identified and codes with similar content and meaning were grouped and organized. The identification of themes and subthemes was conducted by the first author and discussed with all other authors. To optimize rigor and to achieve a proper and rich understanding of the data, further meetings were organized with all authors and the previously consulted researchers involved in the data analysis.

## Results

### Study Characteristics

After the critical selection procedure was completed, the qualitative data from 27 studies with different designs were included: qualitative studies (n = 22; interviews, focus groups, observation, case study designs) and mixed-methods studies (n = 5; survey and interviews, concept mapping, social network analysis and interviews). The studies were conducted in five Western countries: US (n = 15), Australia (n = 5), UK (n = 4), Canada (n = 1), Malta (n = 1), and the Netherlands (n = 1). In the majority of the studies (n = 15), the participants were solely parents – fathers and mothers – of people with ID, but some studies also specifically focused on mothers (n = 3). Some studies included, in addition to parents, other family members as participants, such as siblings (n = 7). Other studies also included, in addition to parents and family members, informal network members from outside the family as participants, such as friends and acquaintances (n = 2). In the majority of the studies, either the level of ID of the relatives was not reported (n = 19) or relatives had various levels of ID (n = 4). A minority of the studies focused on relatives with specific levels of ID: mild (n = 2), severe (n = 1) or visually impaired people with severe or profound (n = 1) ID. In most of the studies, the relatives with ID were either in varying types of employment or daytime activities (n = 15), or there was no specific information given about the type of employment or daytime activities of the relatives (n = 5). A minority of the studies focused on specific types of employment: a day program (n = 1), competitive employment (n = 2), supported employment in the community (n = 2), or self-employed people with ID (n = 2). To improve readability, we use the term “work” to refer to both employment and daytime activities. This is also consistent with the large number of studies that focused on a broad range of work activities and the small number of studies that focused on specific employment types. Finally, the majority of the studies were conducted between 2011 and 2021 (n = 17), eight studies were conducted between 2001 and 2010, and two studies were carried out between 1990 and 2000. The characteristics of all the included studies are shown in Table 2.

**Table 2:** Characteristics of the included studies

No	Author, (year) and country	Aims	Study design, (theory)	Participants	Work situation	Main findings
1	Anderson et al. [38], *US*	Explore early employment-related expectations and experiences through first-person accounts of young adults with autism spectrum disorders (ASD) and their parents	**Qualitative**: Interviews (grounded theory)	**Parents**: fathers, mothers or couples (n = 28) of young adults with ASD and young adults themselves (n = 12). Nine of them had an ID (**level not reported**)	Various work situations from competitive employment without support to day programs	Three major themes emerged: (a) Employment aspirations and potential; (b) Challenges of job finding and keeping; and (c) Differing young adult and parent work-related roles and views.
2	Bianco et al. [39], *US*	Soliciting parents’ voices to provide a better understanding of parents’ experiences and perception of their roles during the post high school years for their children with developmental disabilities	**Qualitative**: semi-structured interviews (phenomenological)	**Parents** (n = 9) of adults with **varying levels of ID**	Working in a vocational setting providing sheltered or supported employment	Families perceived their roles as complex. In advocating for their adult children’s needs into adult life they had to balance between different roles (i.e. collaborators, decision makers, program evaluators, role models, trainers, mentors, instructors, and system change agents). Parents often felt they were the safety net for their children and the back-up plan for service agencies.
3	Butcher & Wilton [37], *Canada*	Exploring the experiences of young adults with ID as they transition from high school in search for paid employment	**Qualitative**: Interviews and participant observations	Young adults with ID (n = 6) (**level of ID not reported**). In addition their **parent(s)** and employer are interviewed.	Various work situations (vocational training center, sheltered workshop and mainstream workplaces)	While the primary goal of the youth and their parents was to make a transition to competitive employment, a lack of transition planning, a shortage of appropriate opportunities and other factors meant the youth spent considerable time in ‘transitional spaces’ (e.g. vocational training center, sheltered workshop, supported employment placements). While these spaces are organized around an explicitly economic goal of augmenting the youth’s employability, they can play a critical role as spaces for social interaction and meaningful activity outside the home.
4	Callus et al. [60], *Malta*	Exploring whether and how people with ID experience overprotection in different aspects of life, especially through the actions of their parents	**Qualitative**: Focus groups	Focus groups with people with ID (n = 17), **parents** of people with ID (n = 14) (**level of ID not reported**) and representatives of organizations (n = 18)	No specific information about work situations	Overprotection is a disabling barrier for employment, leisure activities, intimate relationships, and the use of public transport, money and mobile phones. People with ID who experience overprotection live very structured lives. They cannot develop their skills, abilities, and potential and cannot live their life on their own terms, creating a sustained dependence on others, especially the family.
5	Dague [40], *US*	Examine selected aspects of one agency’s conversion from a sheltered workshop facility to one providing community-based services for individuals with IDD	**Qualitative**: Case study design with semi-structured interviews, participant observation and archival review	People with ID (**level of ID not reported**) and their **parents** (n = 12)	All currently in supported community employment (some transitioned from a sheltered workshop)	While the initial transition was difficult, most families and participants were satisfied with the conversion process as long as they could maintain previous social networks and find acceptable employment in the community. Conflicted issues emerges, as families had different histories, culture, values, philosophies, and expectations of their children and their inclusion in community.
6	Dixon & Reddacliff [45], *Australia*	Describing the contributions families make to the vocational competence of young adults with mild ID	**Qualitative**: Semi-structured interviews	Adults with **mild ID**, in some cases supplemented with interviews with **family members**	Competitive employment	Families contribute to the individuals’ efforts to maintain competitive employment. Moral support, practical assistance, role models of appropriate work ethic, protection from difficulties and exploitation, and family cohesion were family characteristics that led to more successful employment outcomes.
7	Docherty & Reid [48], *UK*	Exploring the values and beliefs of mothers of young adults with Down syndrome (DS), currently involved in supporting their offspring in the transition from dependence to independence	**Qualitative**: Interviews (interpretative phenomenological analysis)	**Mothers** of adults with ID (**level of ID not reported**) (n = 8)	Various work situations	The mothers described themselves as having a dynamic role as both gate-keepers and facilitators in aiding their offspring on the path of adulthood.
8	Donelly et al. [21], *Australia*	Explore the impact of informal support networks on work opportunities of people with ID thoroughly	**Qualitative**: Case-studies with interviews and participant observations (ethnographic)	People with ID (**no level of ID reported**) (n = 4) and key members of their **informal support network**	Various work situations	The insight and actions of network members created and sustained the employment and support opportunities that effectively matched the needs and interests of the relatives with ID. Paid work had a range of personal meanings and functions in the lives of the participants. There was often a mismatch between the interests and choices of the relatives with ID and the services provided by employment support organizations.
9	Eisenman [59], *US*	Further extend the research base on how social networks function to influence careers by examining the experiences of young adults with ID	**Qualitative**: Case-studies with interviews	Adults with **varying levels of ID** (n = 5) and 2 or 3 **informal network members**	Employed (n = 3) or still students (n = 2)	Participants who were still studying mainly had friends and acquaintances from school. Participants who were employed mainly had friends and acquaintances from work. Family members played a supportive role in many aspects of participants’ career situations. Work acquaintances, primarily those in supervising roles, had a strong influence on the career situations of employed participants as did school staff.
10	Finch et al. [46], *UK*	Gain a better understanding of the everyday experiences of autistic adults through first-hand accounts from adults with ASD and also from relatives interviewed from the perspective of supporting autistic adults	**Qualitative**: Interviews (thematic analysis)	Adults with ASD (n = 29) with or without ID **(level of ID not reported)** were interviewed, as well as **relatives** (n = 16): mothers (n = 10), fathers (n = 3), grandparent (n = 1), spouse (n = 1) or sibling (n = 1) of adult with ASD with (n = 9) or without ID	Various work situations	Six main themes were identified: (a) Diagnosis as validating yet limiting; (b) Supportive and non-supportive social agents; (c) The ‘invisibility’ of the needs of autistic adults; (d) Health in the context of autism; (e) Staying ‘outside’ the circle; and (f) Multiple lives with autism. Data from relatives about autistic adults experiences gave additional perspectives on these themes. Education and employment, whilst challenging for many, were also rewarding for some.
11	Ford et al. [49], *Australia*	A preliminary examination of parent and primary caregiver attitudes toward the employment conditions of their relatives/wards who are working in supported employment placements	**Qualitative**: semi-structured interviews	**Parents** (n = 7: 4 mothers, 3 fathers) and professional caregivers (n = 9) of adults with **severe ID**	All in supported employment	Respondents were generally satisfied and accepting of their child’s/ward’s participation in supported employment. They felt that the supported employment programs offered more normalized and beneficial work experiences than those typically provided for persons with severe disabilities. Respondents also expressed relatively low expectations for improving wages, opportunities for career advancement and social integration.
12	Francis et al. [50], *US*	Explore the negative and positive experiences of Hispanic caregivers from a Midwestern state as they support their family members with disabilities to achieve positive post school outcomes	**Qualitative**: Interviews	Hispanic **mothers** (n = 13) of people with various disabilites (age 14–25), including ID (**level of ID not reported**)	Various work situations	Three key themes emerged form analysis: (a) negative experiences with school educators, (b) negative experiences with community-based service providers, and (c) positive experiences and strategies for overcoming barriers.
13	Franklin et al. [41], *US*	Explore the health care transition experiences of parents of adolescents and young adults, with particular interest in the barriers and facilitators to the transition to adulthood within and between the medical, community and vocational systems	**Qualitative**: Semi-structured interviews	**Parents** (n = 16) of adults with **various levels of ID**	Some were in work situations	Three overarching themes represented barriers and the essence of supporting the transition to adulthood of persons with ID experienced by their parents: (a) Inefficient and siloed systems; (b) ‘Left out floundering’ in adulthood; and (c) Hope despite uncertainty.
14	Frounfelker & Bartone [51], *US*	Exploring ways to increase choice for individuals in their day treatment settings and evaluate the effectiveness of a day program, 18 months after its inception, by capturing experiences of participants, family and staff	**Qualitative**: Focus groups (grounded theory)	People with ID (n = 15), **family members** (n = 7), i.e. mothers and sisters, and staff (n = 3), and staff of people with ID (**level of ID not reported**)	All in a day program	A model was derived from the data with ‘building relationships’ at its core. The model describes how relationships come together and work congruently with each level of a day program, from the top administrators to the staff, the members themselves and family members. This helps create and foster a positive environment that increases members’ independence and provides them with stimulating activities during the day.
15	Hall & Kramer [47], *US*	Explore how employment creates opportunities for social capital	**Qualitative**: Interviews and observations	Adults with **various levels of ID** (n = 29), professionals (n = 29) and **family members** (n = 23)	Sheltered workshops and community employment	A form of social capital was created through workplace connections. Community employment did not increase social capital per se, but it did produce opportunities not available in sheltered workshops. The role of family members emerged as critical in the support of community employment, increasing the potential for social capital development.
16	Hanzen et al. [52], *NL*	To develop a definition and operationalization of the concept of participation for adults with visual and severe or profound ID	**Mixed Method**: Concept mapping	**Parents or family members** (n = 30) of people with visual and **severe or profound ID**, professionals (n = 30) and experts (n = 17)	No specific information about work situations	The final cluster map of the statements contained seven clusters: (a) Experience and discover; (b) Inclusion; (c) Involvement; (d) Leisure and recreation; (e) Communication and being understood; (f) Social relations; and (g) Self-management and autonomy. On this basis a definition of participation of the population was developed.
17	Keogh et al. [55], *US*	To document long-term outcomes for individuals with ID specifically to determine where they are and how they are	**Mixed-Method**: Interviews and surveys	**Parents** (n = 30) of young adults with **mild ID**	Various work situations (competitive, assisted, sheltered workshop, volunteering, none)	There was a broad range of outcomes, with some young adults leading independent and productive lives. However, the majority of yound adults were un- or underemployed, living with and financially dependent upon their families, and socially isolated.
18	Lindstrom et al. [54], *US*	Examine career development and early employment experiences of young adults with IDD	**Qualitative**: multiple method, multiple case study design	Adults with ID (**level of ID not reported**) (n = 3) and ASD (n = 1), supplemented with interviews with their **parents**	All in integrated paid employment	During the early career years, participants maintained stable employment, but earned annual wages well under the federal poverty line. Employment opportunities seemed to be influenced by family advocacy and expectations, school-based work experiences, job development services, and work environments.
19	McMahon et al. [57], *Australia*	Explore the aspirations that young adults with intellectual disabilities and their parents hold for the future and the factors that influence their aspirations and decisions regarding support services	**Qualitative**: Interviews (thematic analysis)	Young adults with ID (n = 4; **level of ID not reported**) and their **mothers**	Two young adults were still in education, one was unemployed and one was in supported employment	The four young adults held a range of aspirations themselves. Their mothers had holistic, multidimensional aspirations for their children and wanted them to ‘flourish’ by deriving meaning and enjoyment from employment and leisure activities, having strong supportive relationships with their family, friends and the broader community, and having a happy home environment.
20	Reddington & Fitzsimons [43], *UK*	Identify lessons that could be learned about the role microenterprise might play to help people with learning disabilities to be included in the world of work	**Mixed-methods**: Questionnaire and interviews	Microenterprises (n = 13) were explored, by interviewing persons with ID (**level of ID not reported**), their advocates, **family members** and staff	All in microenterprise	Data reveal how successful self-employment, or starting a microenterprise, has been for the individuals involved, and how this can be a worthwhile alternative to regular employment, training, day center attendance or volunteering.
21	Rossetti et al. [42], *US*	Examine parents’ perceptions of meaningfulness in relation to the young adult’s post-school activities and supports	**Qualitative**: Interviews (Grounded Theory)	**Parents** (n = 23) of adults with pervasive support needs, including ID (**level of ID not reported**)	Diverse work situations (ranging from day program to paid work)	Most of the young adults spent time in their communities, though typically without friends and not engaged in integrated employment. The participants defined meaningfulness according to three dimensions: (a) community participation; (b) individual indicators: and (c) the nature of activities in the young adults’ schedules.
22	Rossetti et al. [58], *US*	Examine parents’ involvement in meaningful post-school experiences	**Qualitative**: Semi-structured interviews	**Parents** (n = 8) of young adults with IDD (**level of ID not reported**)	Various work situations	The parents were active in their children’s post-school experiences as fierce advocates and creative problem solvers. They strove to achieve what they considered to be the most meaningful lives for them, describing that they often challenged existing service options to do so. The active involvement of parents included three participatory sub-themes, which contributed to their success: (a) attitudinal factors, (b) advocacy efforts, and (c) strategic actions.
23	Spencer et al. [56], *US*	Provide insights into the social networks of graduates of a post-secondary education program and the social network aspects that contribute to positive employment outcomes	**Mixed-methods**: Semi-structured interviews and social network analysis	Adults with ID (n = 6) (**level of ID not reported**) and their **parents/guardians** (n = 6)	Five participants were employed, one had recently become unemployed	Three participants displayed smaller networks than a year before. Two graduates displayed larger networks, because of new opportunities for socialization. Most parents were involved in graduates’ employment decisions, thereby curbing graduates’ expression of self-determination.
24	Thomson et al. [53], *UK*	Reporting on the transition pathways to the adult status of young adults with DS	**Mixed-Method**: Quantitative and qualitative (case study) data	Adults with DS (**level of ID not reported**) (n = 2). In the case-studies the **parent** perspective is described	day centre (n = 1) and volunteering (n = 1)	Results provide a disappointing picture of a narrow range of leisure opportunities, negligible placement in employment, and continuing dependency on parental caregivers in adulthood.
25	Thoresen et al. [35], *Australia*	Explore the utility of Small Business Enterprises (SBE) as an emerging employment pathway in economic and social inclusion for adults with ID	**Qualitative**: Case study design with interviews	Case studies of people with ID and high support needs (**level of ID not reported**) (n = 4) that also includes the perspective of **parents** as key informants	All self-employed	A range of strategies are described to develop and maintain SBE’s and to create meaningful economic and social inclusion. The case studies illustrate different supports used in establishing and continuing these arrangements, and issues related to safeguarding and sustainability.
26	Timmons et al. [44], *US*	Exploring factors that influence the employment-related decision-making of individuals with IDD and to what extent their preferences correspond to existing employment options and choices	**Qualitative**: Interviews	Adults with ID (**level of ID not reported**) (n = 16), and one of their **family members** and professional caregivers	Sheltered (n = 7) and competitive (n = 9) employment	Different people and factors affect the choices people with IDD make in relation to work: (1) family in the formative years, (2) school-based staff and early employment experiences, (3) community rehabilitation providers’ culture, (4) the job developer and, (5) personal preferences.
27	Yuan et al. [36], *US*	Explore parents’ expectations and perceptions of academic, social and employment experiences of their children out of the first two cohorts of the Think College	**Qualitative**: Semi-structured interviews	**Parents** (n = 9) of adults with ID (**level of ID not reported**)	Majority in paid employment	Parents described efforts to support their children during college and discussed hopes for employment, and evolving perceptions of their own roles in relation to the future lives of their adult children. Parents responses fell into three categories: (a) perception of their child, (b) perceptions of the program (including expectations and perceptions of employment), and (c) perceptions of their own roles of the college life of their children and beyond.

### Thematic Synthesis

Thematic synthesis yielded four overarching themes with various subthemes, which are discussed in turn below. An overview of the reported themes and subthemes for each study is presented in Table 3.

**Table 3:** Themes and quality assessment per study

Study	I: Ensuring customized work for my relative		II: My ongoing need to collaborate and share care responsibilities	III; The meaning of participation in work for both my relative and myself				IV: Achieving full work participation for my relative is neither straightforward nor self-evident	
	a): My efforts to put the wishes and needs of my relative first	b): A lack of support services and work options that fit my relatives’ needs		a): Work provides routine and purpose in my relative’s life	b): Work helps my relative to keep developing skills and feel self-confident	c): Employment improves my relatives’ social connections in the community	d): I want my relative to make a contribution to society and perform a valued social role	a): Moving a continuum: Ideals for participation in community jobs vs. barriers and concrete experiences in society	b): Basic income insecurity hinders my relative’s full participation in the workforce
Anderson et al. [38]***		x	x	x	x	x	x	x	
Bianco et al. [39]***		x	x		x			x	
Butcher & Wilton [37]*	x	x	x	x		x	x	x	
Callus et al. [60]*								x	
Dague [40]**	x	x	x	x	x	x	x	x	
Dixon & Reddacliff [45]***			x					x	
Docherty & Reid [48]**			x		x				
Donnelly et al. [21]***	x	x	x	x	x		x	x	x
Eisenman [59]***			x					x	
Finch et al. [46]***		x	x	x				x	
Ford et al. [49]***	x	x	x	x	x		x	x	x
Francis et al. [50]***		x	x			x		x	x
Franklin et al. [41]***		x	x	x	x	x		x	
Frounfelker & Bartone [51]***	x	x	x	x	x			x	
Hall & Kramer [47]**		x	x					x	x
Hanzen et al. [52]***	x			x	x	x	x	x	
Keogh et al. [55]**		x			x				
Lindstrom et al. [54]***	x		x		x	x		x	x
McMahon et al. [57]**	x		x	x				x	
Reddington & Fitzsimons [43]*		x							x
Rossetti et al. [42]**	x	x	x	x	x		x	x	
Rossetti et al. [58]***				x				x	
Spencer et al. [56]***			x		x	x		x	x
Thomson et al. [53]*					x			x	
Thoresen et al. [35]*	x	x	x	x	x	x	x	x	
Timmons et al. [44]***		x	x	x			x	x	
Yuan et al. [36]***	x	x						x	x

* Low quality score on MMAT.

** Medium quality score on MMAT.

*** High quality score on MMAT.

*MMAT* Mixed-Method Appraisal Tool

### Theme I: Ensuring Customized Work for my Relative

Across the studies, informal network members expressed the importance of ensuring customized work for their relatives with ID. Within this theme, two subthemes emerged: One concerning the efforts of network members to put the needs and wishes of their relatives first in the process of finding or creating customized and person-centered work for them; and one centered on network members’ experiences of the lack of support services and work options that suit their relatives’ needs.

#### My Efforts to put the Wishes and Needs of my Relative First

Informal network members went to considerable effort to find or create work activities that their relatives preferred, in addition to work activities that suited the needs and skills of their relatives. They emphasized the value of focusing on the wishes, dreams and needs of their relative as the starting point for creating meaningful employment for them (n = 11). Parents, for example, stressed the importance of putting their child’s interests at the center of starting up a Small Business Enterprise (SBE) [35]. One parent stated: *“The potential for other people to create person-centered employment is limitless, it’s absolutely limitless. It just had to be looking at who is the person, what can they do, what do they want, what are they like?”* In addition, network members stressed that leveraging their knowledge about the interests, skills and choices of their relatives with ID served as an effective basis from which to create employment opportunities, identify support needs and find ways to effectively meet those needs [21].

In some instances, it appeared to be complex for parents to distinguish between their child’s wishes and their own wishes for them. For example, a parent couple expressed that they were actively trying not to determine the direction their child should take [36]. Moreover, in some of the cases from the study by Butcher & Wilton [37], the preferences of the relatives with ID interfered with the expectations and preferences of the parents. For example, one mother felt the need for her son to do a job that would be more challenging for his brain and considered him capable of that, despite knowing that her son liked his current job in a grocery store.

#### A Lack of Support Services and Work Options that Fit my Relatives’ Needs

Despite the fact that informal networks emphasized the importance of customized work for their relatives with ID, they indicated that existing customized work opportunities were limited, and that there was a lack of support in finding suitable options (n = 17). It was common for informal network members to spend a lot of time and effort trying to obtain suitable support and work opportunities, and sometimes they had to endure years of waiting lists for support services and work. This frequently resulted in network members having to strongly advocate for their relatives with ID to obtain support and work [21, 35, 37–42]. Parents, for example, experienced having to fight to achieve employment or the necessary support for their children [39]. In the absence of existing suitable programs or because of the unavailability of programs due to long waiting lists, parents in some cases initiated suitable work opportunities for their relatives themselves, by creating micro-businesses [35, 42, 43], collaborating to establish day programs [21, 37, 40], or arranging volunteer opportunities [38].

### Theme II: My Ongoing need to Collaborate and Share Care Responsibilities with Professionals

Informal networks reported the need to organize sustainable work for their relatives with ID in sustainable collaboration with professionals (n = 20). For example, network members emphasized that a sustainable and ongoing supportive partnership with a support worker was a precondition for sustainable work for their relatives with ID, rather than forms of support that were time-limited and focused on withdrawal [21]. However, informal network members sometimes had different expectations about the allocation of roles and tasks between network members and professionals. For example, family members perceived support workers, such as job coaches, to have greater knowledge and skills than themselves about finding work for their relatives [44]. They viewed job coaches as being responsible for finding and preparing their child for starting work. In other studies, the network members reported playing a more active role in the work support for their relatives [21, 38, 39, 45–47]. For example, in the study of Bianco et al. [39], parents took on the role of teaching their children job-related skills. One mother, however, experienced that this role of being a mother and a job coach simultaneously eventually became unwieldy for her, and she chose to hand over that role to a professional. She expressed: *“I was the main support [for a job] and I’m also the main reason why we’re not doing it still. Because it became unwieldy and I did not have enough time to be that support person.”*

With respect to their collaboration with professionals and other people, such as employers and co-workers of their relatives with ID, informal network members expressed the need for shared responsibility [21, 37, 39–42, 48–50]. If and when network members felt they were able to confidently share the care and responsibilities for their relatives with ID, it brought them relief and comfort. Indeed, even the fact that their relatives with ID had a job was in some instances experienced in and of itself as a form of support by network members. Involvement in work activities made network members feel that their relatives were supervised for a certain number of hours during the week [21, 37, 38, 40, 45, 49, 51]. In some instances, parents explicitly expressed that the work of their child released them from their caregiver duties and freed them up for their own schedules and interests [40, 49].

### Theme III: The Meaning of Work for Both my Relative and Myself

It was observed across the studies that informal networks experienced work as being meaningful for their relatives with ID, in terms of providing several benefits in both their relatives’ and sometimes their own lives, namely: (I) gave them routine and purpose, (II) enhanced their skill development and self-confidence, (III) provided them with social connections, and (IV) afforded them opportunities to contribute to society and perform social roles.

#### Work Provides Routine and Purpose in my Relative’s life

Although a few parents (Rossetti et al., 2015; 2016) stated that they experienced life outside of work as more meaningful for their child, involvement in work was generally perceived as a means for the relatives with ID to spend the day performing meaningful activities (n = 14). For example, parents experienced that it provided routine [35, 37, 40] or structure [38, 46] and a sense of purpose [35, 42, 51] in their relatives’ lives. In some studies, family members noted that variation in work activities was important for experiencing meaningfulness.

Furthermore, in several studies informal networks reported negative effects of the absence of work for their relatives with ID [21, 37, 41, 42, 49, 51, 52]. For example, network members experienced that days without work could be very long and boring for their relatives. They reported that long periods without work could also have adverse health effects for them, for example weight gain and depression [21]. Parents indicated that a lack of structure and meaningful daytime activities negatively impacted on the situation of their relatives, as a result of reversing the day-night rhythm [37]. In some instances, this also had negative effects upon family members. One mother experienced significant conflict and role confusion, because her daughter was at home every day, and she considered this to be detrimental to both her daughter and the family. She said: *“I’m just trying to find something for [my daughter] to do because it is certainly detrimental to her and to everybody else that she is home every day. (…) because it gets where she is trying to reverse roles. She is trying to be the mother and telling you know, what to do, what to wear. There’s been a fair amount of conflict with her and I lately…”*.

#### Work Helps my Relative to Keep Developing Skills and Feel Self-Confident

Informal network members believed that participation in work contributed toward the development of their relatives’ skills and self-confidence (n = 15). For example, one father noted that his daughter’s self-confidence had markedly increased since she started attending a day center [53]. In some of the studies, network members associated the development of skills and self-confidence particularly with work in the community [21, 35, 40, 54]. Dague’s study [40], for example, explored how parents experienced the transition from a sheltered workshop to community employment, with several of them reporting a remarkable increase in their children’s skills since leaving the sheltered workshop, including their verbal communication skills. In the study of Donnelly et al. [21], the opportunities that community employment afforded to develop traveling and social skills were valued. Conversely, a family member whose relative had returned to sheltered employment experienced that this relative was no longer challenged at work and consequently saw their skill diminish.

Nevertheless, parents sometimes felt that there were insufficient career opportunities for their relatives to continue to develop their skills. One father, for example, noted that there were no actual further positions his son could grow to occupy, with the exception of supervisor positions that he personally felt would be too demanding in view of his son’s capabilities [42, 49, 55, 56]. In his words: *“There is nowhere to go except maybe a supervisor position, but [son] doesn’t have the ability for that.”*

#### Employment Improves my Relatives’ Social Connections in the Community

Informal networks experienced that work participation improved their relatives’ social connections in the community, beyond their families (n = 9). One mother, for example, expressed that she felt it was crucial that work in the community made her son meet people besides his own family [54]. She said: *“He sees a whole different world of people besides family and it’s just crucial to his whole [being]. It seems the healthy thing to do for any adult with a disability.”* Another mother noticed that her son developed relationships with his co-workers, who started monitoring and helping him outside of working hours, as well as including him in social events outside work [50]. Some parents found it very difficult that their child had to make the transition from a sheltered workshop to work in the community [40]. They wanted to maintain the sense of community and connection that had developed in the sheltered workshop over 35 years and were concerned about losing this, not only for the sake of their relatives with ID, but also for their families. They experienced a sense of togetherness with other parents and feared the loss of these friendships among families.

#### I Want my Relative to Make a Contribution to Society and Perform a Valued Social Role

Participation in employment was perceived by informal networks as a means through which their relatives with ID could make a productive contribution to and perform a valued social role in society (n = 9). Besides making a productive contribution, network members considered it as meaningful when their child could make a difference to other people’s lives and receive recognition for this [21, 40, 42]. One mother, for example, expressed that her daughter was recognized for her ability to help day care children go to sleep [40]. She said: *“She’s the lunch lady there. She does the tables, cleans them all up, pours the milk, sweeps the flours, takes the garbage out, and she’s been known to put twenty children on the back and put them all to sleep there.”*

However, making a contribution to society was not only perceived as being valuable for their relatives with ID, rather it also brought relief to family members [35, 49]. Parents experienced a sense of relief as their children became productive members of society [49]. For example, one mother felt less guilty since her son was involved in meaningful work activities, as opposed to before when he was just sitting at home and benefitting from society. She even felt that in a similar way other family members’ previous feelings of guilt had been replaced by pride in her son’s achievements.

### Theme IV: Achieving Full Work Participation for my Relative is Neither Straightforward Nor Self-evident

Although many informal network members wanted their relatives with ID to be able to experience full participation in society via work, they also experienced many societal barriers that hindered this.

#### Moving a Continuum: Ideals for Participation in Community Jobs vs. Barriers and Concrete Experiences in Society

Many network members in the studies were focused on achieving a full job and experiencing a sense of belonging to society for their relatives with ID (n = 25). They routinely expressed a strong preference for community jobs and valued normalization for their relatives [21, 35, 37–40, 44, 47, 49–51, 57, 58, 59]. Mothers, for example, wanted to ensure that their children with ID felt integrated in society like any other individual [50]. They believed that they themselves, as parents, could help make this happen by maintaining high expectations and a positive outlook on participation.

Furthermore, network members in some instances even expressed strong disapproval toward work in sheltered environments [21, 37, 40, 49]. For example, some parents considered working in a sheltered workshop as working in a closeted box, saw sheltered workshop tasks as both menial and redundant or thought that working in a sheltered workshop added to the stigma toward people with ID [40]. Moreover, there were concerned that placement in sheltered employment would limit the future opportunities of relatives with ID to grow into competitive employment [21, 37]. Informal networks, however, encountered obstacles in realizing their aspirations for participation. Indeed, sometimes it took considerable effort to achieve participation in community jobs [21, 37–39].

Conversely, there were family members who expressed a clear preference for sheltered work environments [21, 40, 45, 47, 51, 60]. These family members expressed concerns about unfair treatment and wanted to protect their vulnerable relatives from exclusion, negative experiences, and high demands in community jobs. There were fears and concerns about exploitation of their relatives with ID by employers or co-workers, by, for example, assigning them too heavy tasks or by asking them to borrow money, or about bullying by co-workers, discrimination or dismissal from jobs on the grounds of their disability [37, 40, 45, 60]. Therefore, some parents felt that their children were better off in sheltered employment situations or staying at home instead of doing community jobs [60].

However, the preferences of network members for community or sheltered work sometimes changed over time. These changes might have been driven by advanced insights through experiences or observations of their child’s experiences and abilities. For example, parents who feared the closure of the sheltered workshop in the study of Dague [40] reported after four years that their initial fears of abuse and ridicule in a community job did not materialize. Rather, they experienced that community-based jobs were tailored to the needs of their children and caused their children to be considered ‘regulars’ and be recognized in the community. Conversely, one mother, who initially feared the placement of her daughter in a sheltered workshop, saw after six months of the placement the various benefits of the sheltered workshop for her daughters’ wellbeing, as well as the risks and barriers of working in the community [37]. She subsequently abandoned her ideal for participation in society and changed her belief in seeing separation as positive. Moreover, the study of Dague [40] indicated that the preferences of network members for sheltered vs. community work might also derive from the spirit of the time in which their relatives with ID had grown up. Some parents thought it was too late for their children with ID to make the shift from the sheltered workshop to community employment. These parents had noticed that people with ID today are better prepared and educated for community jobs, while their children did not have that option during their younger years. One mother thought it was unfair to expect her child to go into a community job, because her child did not know any better. She said: *“We didn’t prepare them for this. The younger kids have the potential, our kids don’t. They have been too taken care of. It’s not fair to them.”*

#### Basic Income Insecurity Hinders my Relative’s full Participation in the Workforce

Across several of the studies, informal networks expressed concerns about the effects of full participation in paid employment upon their relatives’ eligibility for social benefits and the financial risks this entailed (n = 8). As a result, network members experienced struggles and sometimes had to make paradoxical decisions in order to protect the financial situation and social benefits of their relatives.

Network members in the study of Donnelly et al. [21], for example, experienced a huge struggle, as their relative worked far too few hours, purely due to the risk of losing her benefits. They experienced a paradox, insofar as working the desirable number of additional hours represented a potential threat to the social security of their relative. Family members in other studies also took paradoxical decisions about the work situations and earnings of their relatives [36, 47, 54]. For example, parents decided to limit their son’s working hours to protect his benefits, despite having an offer for full-time paid employment. They were concerned that he would not recover his benefits when he lost his job and feared financial hardship, with a view to their own mortality [36]. Intense fear over losing benefits led some parents to suggest that their children should work without getting paid [47], while other parents protected their children’s benefits by instructing employers to pay their children low wages. As one mother said: *“I had said to [his supervisor] don’t pay him much now because I don’t want to go over that limit and go through that again….and [his job coach] did say he can’t make too much money….”*

## Discussion

The present study systematically reviewed qualitative data from qualitative and mixed-methods studies (n = 27) to explore how informal networks of adults with ID perceive and experience the meaning of participation in work for their relatives with ID. The thematic synthesis revealed four overarching themes: (I) Ensuring customized work for my relative; (II) The ongoing need to collaborate and share care responsibilities with professionals; (III) The meaning of work for both my relative and myself; and (IV) Achieving full work participation for my relative is neither straightforward nor self-evident. The results suggest that informal network members place significant value on the customization and maintenance of work opportunities for their relatives with ID, particularly community-based work.

The first two themes of the present review revealed that in finding sustainable and fitting work, informal networks considered it an important precondition to always position the preferences, skills and needs of their relatives with ID at the forefront of their choices. However, informal network members experienced a lack of support services and work opportunities that matched their relatives’ preferences, skills and needs, which, in turn, hindered the needed customization. Network members believed that realizing sustainable work that matches the preferences, skills and needs of their relatives with ID, requires ongoing collaboration with professionals, employers and co-workers. In their estimation, this involves sharing responsibilities and considering the informal network as a partner, which is in line with previous research findings [22]. However, network members experienced two types of problems in creating this partnership in practice. On the one hand, network members experienced that their knowledge of their relatives’ preferences, skills and needs is sometimes underutilized by professionals. On the other hand, in some instances too many tasks, such as job coaching, fall on the shoulders of network members, which can overburden them or cause confusion about their role as either a loved one or a professional. Nevertheless, if collaboration and shared responsibility between professionals and informal network members are well-balanced, then this can promote the resilience of informal networks [61]. Petner-Arrey et al. [22] also found that continued advocacy for and investment in supporting informal networks to achieve suitable work for their relatives with ID could lead them to experience fatigue and frustration. Therefore, it is recommended that professionals and employers take note of the experiential knowledge of network members of people with ID, seek to utilize their experiential knowledge and collaborate with them in partnership. It is equally important for professionals to be sensitive to the capacity and workload of network members in the process of securing work for their relatives with ID. Professionals might consider whether, if necessary, they can take over certain tasks from network members and, particularly in the case of parents, help strengthen the support from the social network [22]. Given the growing recognition of the importance of experiential knowledge of informal networks of people with ID in terms of collaborating with professionals and engaging in shared decision-making [62], it is important that research continually acknowledges the voices of informal networks.

The third theme showed that informal network members experienced suitable work to be meaningful for their relatives, and in some instances also for themselves and the broader social network. Most of the benefits attributed to work by informal networks were related to their relatives’ position in society. They experienced that skill development and self-confidence were positively associated with work in the community. Moreover, they valued the way in which work increased their relatives’ social connections and social capital through affording them contact outside of their family and immediate environment, and, in so doing, enabled them to contribute to society through either their productivity or by doing something meaningful for other people. These perceptions of informal networks are consistent with how people with ID themselves experience the meaning of work [7, 18] as well as how those without ID perceive work [63]. However, the difficulties that the informal networks in this study experienced in ensuring community-based work may hinder people with ID from benefitting from work as the primary means through which to experience social connections, inclusion, feelings of belonging, and social relevance [6].

The fourth theme revealed that, consistent with the benefits of work experienced by informal network members, many of them had a clear preference for community-based work and the associated normalization and equality. Indeed, in some instances they even expressed strong disapproval toward sheltered work. Other network members, however, preferred sheltered work because of the risks and dangers they perceived in society. This difference touches upon the ongoing debate in the field of ID about the appropriate balance between protection and empowerment [2]. Informal networks of people with ID have to strike a balance between protection and empowerment when supporting their relatives. The findings further revealed that network members’ preferences for either sheltered work or work in the community may change over time, as a result of their experiences or due to societal developments and shifts in prevailing norms and values, the related societal image of people with ID and thus the prevailing public stigma [64]. As a result, network members could potentially experience courtesy stigma, even though they did not explicitly report it in the studies. Courtesy stigma occurs when the stigma also affects persons closely related to the stigmatized person [65]. This, in turn, can impact upon the wellbeing of network members, especially when courtesy stigma leads to the development of negative self-evaluations and negative emotions, which is referred to as affiliate stigma [65, 66]. Given that self-esteem and social support are found to be potential buffers against the internalization of stigma for network members [66], this further emphasizes the need to strengthen the support from their social network. To strengthen the social network, professionals can, for example, use the strategy of Family Group Conferencing [67, 68]. However, future research is needed to better understand the effect of (courtesy) stigma on informal network members of people with ID.

The findings point to informal networks experiencing one clearly distressing barrier: the potential impact of work participation on their relatives’ basic income (in)security. Network members in studies from the US, Australia and the UK report on this issue, with specific experiences varying depending on different national social security systems. These experiences sometimes forced network members to make paradoxical decisions regarding their relative’s work, such as, for example, advising them to turn down an offer for a full-time job. In so doing, they unintentionally reproduced the deficient work participation of people with ID. This finding indicates that legislation and social security systems are not in line with the participation and autonomy of people with ID. Moreover, this is an example of structural stigma, which is ingrained in the political system and hinders people with ID from fully participating in society [69, 70]. It raises the question of to what extent the ideals of ‘independence’, ‘autonomy’, ‘citizenship’, and ‘inclusion’ actually fit the way people with ID are viewed by the general population [2, 71], and even care professionals [70], in a performance-oriented system defined by ableism [69]. Reforming these types of systems is necessary for overcoming structural stigma, starting with a recognition by public institutions of the deleterious impact that structural stigma can have on the well-being of people with ID as well as their livelihood security [69]. In collaboration with people with ID, their networks, and experts-by-experience, policy makers can find approaches to adopt legislation in such a way that promotes full work participation by people with ID, while, simultaneously, providing them with a financial safety net for when they are unable – temporarily or for an extended period – to engage in paid work. Ideas of ‘contributive justice’ rather than merely ‘distributive justice’ can be involved in these new approaches to creating work opportunities for everyone, thus allowing society to make use of everyone’s talents rather than providing benefits [72].

This thematic synthesis of qualitative data makes an important scientific contribution by bringing together extant knowledge about informal network members’ perspectives toward work for their relatives with ID. Specifically, by applying thematic synthesis, the perspectives of participants from a range of study contexts can be examined both deeply and broadly, thus providing evidence for the development and implementation of interventions [73]. However, consideration should also be given to the limitations of this synthesis. First, some of the studies (n = 5; 19%) in this synthesis received low quality ratings. The results of these studies may therefore have a lower reliability. However, both the considerable number of studies included in this synthesis (n = 27) and the good representation of these studies within the themes and subthemes found, may have positively affected the reliability of the synthesis. Next, although this study focused on informal networks, most of the included studies appeared to address the perspective of parents of people with ID, which is in line with the fact that most of the practical and emotional support provided to people with ID comes from their parents [24, 27]. The results should therefore be interpreted accordingly. Future empirical studies could focus on the perspective of other network members, such as siblings, friends, and acquaintances, in order to gain more insight into the potential role of peer groups in finding and retaining work for people with ID. The ways in which the informal networks of people with ID contribute to finding and retaining work could also be compared to the way informal networks and reference systems of the general population function in the labor market, from the viewpoint of labor market and job search theory. A further limitation pertains to the fact that all the studies were conducted in Western countries, with the vast majority being from the US (n = 15, 56%). Consequently, the results of this synthesis cannot be directly generalized to non-Western contexts or even to European welfare states. Moreover, the specific context should be considered at all times when interpreting the results. One of the studies was conducted in Malta [60], explicitly stating that protection is characteristic of Maltese culture. Indeed, the results of this study reveal more (over)protection by informal network members than the results from other studies.

This thematic synthesis enhances our understanding of the value of work for individuals with ID through the lens of informal networks, highlighting the important role that network members play in creating sustainable and appropriate work opportunities for their relatives. This deeper understanding may inspire researchers, professionals, policy makers, and employers to strengthen collaboration with individuals with ID as well as their networks to increase the meaningful work opportunities available to them.

### Electronic Supplementary Material

Below is the link to the electronic supplementary material.

Supplementary Material 1
