# Supplementary material for: Informal Network Members’ Perspectives and Experiences on Work for People with Intellectual Disabilities: A Thematic Synthesis
Source: J Occup Rehabil. 2023 Jul 8;34(1):37–55. doi: 10.1007/s10926-023-10128-0 (PMC10899270; doi:10.1007/s10926-023-10128-0)
Supplement: Supplementary file 1 — Supplementary Material 1 [file 10926_2023_10128_MOESM1_ESM.docx]

Appendix I: Full search strategy

Embase

('intellectual impairment'/de OR 'mental deficiency'/exp OR 'learning disorder'/de OR 'mentally disabled person'/de OR 'developmental disorder'/de OR (((intellect* OR learning OR development*) NEXT/1 (defect* OR deficit* OR deficien* OR dysfunction* OR disab* OR impair* OR disorder* OR retard* OR handicap*)) OR ((mental*) NEXT/1 (defect* OR deficit* OR deficien* OR dysfunction* OR disab* OR impair* OR handicap* OR retard*)) OR retard OR retarded OR retards OR idiocy OR (down* NEAR/3 syndrome*) OR prader-willi OR fragile-x):ab,ti,kw) AND ('employment'/exp OR 'sheltered workshop'/de OR 'volunteer'/exp OR 'vocational rehabilitation'/de OR vocation/de OR 'vocational guidance'/de OR occupation/de OR 'job performance'/de OR 'job satisfaction'/de OR 'productivity'/de OR 'quality of working life'/de OR 'work experience'/de OR 'workplace'/de OR work/mj OR (employ* OR unemploy* OR ((sheltered* OR protected) NEXT/1 work*) OR volunteer* OR vocation* OR Day-habilitation OR Day-activit* OR Daycent* OR Day-cent* OR Employ* OR Entrepreneur* OR Job OR jobs OR Occupation* OR (work* NEXT/2 (inclusi* OR integra* OR particip* OR rehabilitation OR status OR productivit* OR placement OR performance* OR condition* OR satisf* OR experience*)) OR paid-work OR Workplace OR Work-place OR coworker* OR co-worker* OR hiring):ab,ti,kw OR work:ti) AND ('family'/exp OR 'informal caregiver'/de OR 'mentor'/de OR 'family attitude'/exp OR caregiver/de OR (family OR families OR Acquaintance* OR Brother* OR Companion* OR Daughter* OR Friend OR Friends OR Grandfather* OR Grandmother* OR Grandparent* OR Husband* OR Maternal OR Mentor OR Mentors OR Mother* OR Father* OR Parent* OR Partner* OR Paternal OR Relatives OR Sister* OR Son OR sons OR Stepparent* OR Stepfamily OR Stepfather* OR Stepmother* OR Wife* OR caregiver*):ab,ti,kw) NOT ([Conference Abstract]/lim) NOT ((animal/exp OR animal*:de OR nonhuman/de) NOT ('human'/exp)) AND [English]/lim

Medline

(exp Intellectual Disability / OR Developmental Disabilities / OR Learning Disorders / OR Mentally Disabled Persons / OR (((intellect* OR learning OR development*) ADJ (defect* OR deficit* OR deficien* OR dysfunction* OR disab* OR impair* OR disorder* OR retard* OR handicap*)) OR ((mental*) ADJ (defect* OR deficit* OR deficien* OR dysfunction* OR disab* OR impair* OR handicap* OR retard*)) OR retard OR retarded OR retards OR idiocy OR (down* ADJ3 syndrome*) OR prader-willi OR fragile-x).ab,ti,kw.) AND (Employment/ OR Sheltered Workshops/ OR Volunteers/ OR Rehabilitation, Vocational/ OR Vocational Guidance/ OR Occupations/ OR Work Performance/ OR Job Satisfaction/ OR Workplace/ OR *Work/ OR (employ* OR unemploy* OR ((sheltered* OR protected) ADJ work*) OR volunteer* OR vocation* OR Day-habilitation OR Day-activit* OR Daycent* OR Day-cent* OR Employ* OR Entrepreneur* OR Job OR jobs OR Occupation* OR (work* ADJ2 (inclusi* OR integra* OR particip* OR rehabilitation OR status OR productivit* OR placement OR performance* OR condition* OR satisf* OR experience*)) OR paid-work OR Workplace OR Work-place OR coworker* OR co-worker* OR hiring).ab,ti,kw. OR work.ti.) AND (exp Family/ OR Mentors/ OR Caregivers/ OR (family OR families OR Acquaintance* OR Brother* OR Companion* OR Daughter* OR Friend OR Friends OR Grandfather* OR Grandmother* OR Grandparent* OR Husband* OR Maternal OR Mentor OR Mentors OR Mother* OR Father* OR Parent* OR Partner* OR Paternal OR Relatives OR Sister* OR Son OR sons OR Stepparent* OR Stepfamily OR Stepfather* OR Stepmother* OR Wife* OR caregiver*).ab,ti,kw.) NOT (news OR congres* OR abstract* OR book* OR chapter* OR dissertation abstract*).pt. NOT (exp animal/ NOT human/) AND english.la.

PsycINFO

(exp Intellectual Disability / OR Developmental Disabilities / OR Learning Disorders / OR Mentally Disabled Persons / OR (((intellect* OR learning OR development*) ADJ (defect* OR deficit* OR deficien* OR dysfunction* OR disab* OR impair* OR disorder* OR retard* OR handicap*)) OR ((mental*) ADJ (defect* OR deficit* OR deficien* OR dysfunction* OR disab* OR impair* OR handicap* OR retard*)) OR retard OR retarded OR retards OR idiocy OR (down* ADJ3 syndrome*) OR prader-willi OR fragile-x).ab,ti.) AND (Employment/ OR Sheltered Workshops/ OR Volunteers/ OR Rehabilitation, Vocational/ OR Vocational Guidance/ OR Occupations/ OR Work Performance/ OR Job Satisfaction/ OR Workplace/ OR *Work/ OR (employ* OR unemploy* OR ((sheltered* OR protected) ADJ work*) OR volunteer* OR vocation* OR Day-habilitation OR Day-activit* OR Daycent* OR Day-cent* OR Employ* OR Entrepreneur* OR Job OR jobs OR Occupation* OR (work* ADJ2 (inclusi* OR integra* OR particip* OR rehabilitation OR status OR productivit* OR placement OR performance* OR condition* OR satisf* OR experience*)) OR paid-work OR Workplace OR Work-place OR coworker* OR co-worker* OR hiring).ab,ti. OR work.ti.) AND (exp Family/ OR Mentors/ OR Caregivers/ OR (family OR families OR Acquaintance* OR Brother* OR Companion* OR Daughter* OR Friend OR Friends OR Grandfather* OR Grandmother* OR Grandparent* OR Husband* OR Maternal OR Mentor OR Mentors OR Mother* OR Father* OR Parent* OR Partner* OR Paternal OR Relatives OR Sister* OR Son OR sons OR Stepparent* OR Stepfamily OR Stepfather* OR Stepmother* OR Wife* OR caregiver*).ab,ti.) NOT (news OR congres* OR abstract* OR book* OR chapter* OR dissertation abstract*).pt. NOT ((animal.po. OR exp animals/) NOT human.po.) AND english.la.

Web of Science Core Collection

TS=(((((intellect* OR learning OR development*) NEAR/1 (defect* OR deficit* OR deficien* OR dysfunction* OR disab* OR impair* OR disorder* OR retard* OR handicap*)) OR ((mental*) NEAR/1 (defect* OR deficit* OR deficien* OR dysfunction* OR disab* OR impair* OR handicap* OR retard*)) OR retard OR retarded OR retards OR idiocy OR (down* NEAR/2 syndrome*) OR prader-willi OR fragile-x)) AND ((employ* OR unemploy* OR ((sheltered* OR protected) NEAR/1 work*) OR volunteer* OR vocation* OR Day-habilitation OR Day-activit* OR Daycent* OR Day-cent* OR Employ* OR Entrepreneur* OR Job OR jobs OR Occupation* OR (work* NEAR/2 (inclusi* OR integra* OR particip* OR rehabilitation OR status OR productivit* OR placement OR performance* OR condition* OR satisf* OR experience*)) OR paid-work OR Workplace OR Work-place OR coworker* OR co-worker* OR hiring) OR work:ti) AND ((family OR families OR Acquaintance* OR Brother* OR Companion* OR Daughter* OR Friend OR Friends OR Grandfather* OR Grandmother* OR Grandparent* OR Husband* OR Maternal OR Mentor OR Mentors OR Mother* OR Father* OR Parent* OR Partner* OR Paternal OR Relatives OR Sister* OR Son OR sons OR Stepparent* OR Stepfamily OR Stepfather* OR Stepmother* OR Wife* OR caregiver*))) AND DT=(article OR review OR Letter OR Early Access) AND LA=(english)
